# The Status of Cocaine in Surgery

**Published:** 1889-12

**Authors:** John A. Wyeth

**Affiliations:** New York


### Journal Abstracts.

### THE STATUS OF COCAINE IN SURGERY.

By JOHN A. WYETH, M. D., New York.

The *International Journal of Surgery*, for October, 1889, contains a useful article by Professor John A. Wyeth, of New York, upon "The Status of Cocaine in Surgery." After dwelling briefly upon the history of cocaine, and eulogizing Koller, the demonstrator of its anesthetic properties, the author introduces his subject by alluding to the dangers of the drug. Idiosyncrasy is first mentioned as accounting for some of the accidents which have occurred. To avoid such occurrences, "the general rule should be, to begin with the minimum, gradually increasing as slowly as possible, always watching the pulse, the face, the respiration, and the pupil." The dosage varies in different individuals, and in the same individual at different times. It should never be given to children under ten or twelve years of age. In these latter cases the author thinks that the sight of the blood and wound, and the excitement of the operation, sometimes cause the unpleasant symptoms, but not always, absorption of the drug being a cause as well.

Absorption is characterized by various symptoms, exhilaration, convulsive movements, dilatation of the pupil, and increased heart action.

Professor Wyeth prefers a four per cent. solution hypodermatically. A handy formula, approximately four per cent., runs thus: Distilled water, one ounce; cocaine hydrochlorate, grains twenty, and boracic acid, one to three grains.

The technique of operations upon the extremities is thus described, amputation at the last interphalangeal joint of the finger being taken as an example: "The hand is cleansed by immersion in 1-2000 sublimate solution for half an hour. The anesthetic may be employed in two ways, viz., *directly, injected in the lines of incision, or indirectly, injected above the nerves at the base of the finger.*" In the author's opinion, cocaine directly injected retards, to a slight degree, union and repair in wounds. A hypodermic syringe, with delicate and clean needles, is selected, the quantity of cocaine accurately measured, the screw on the piston being run down to prevent the injection by accident of more than a few minims. The finger is constricted by means of a piece of rubber tubing around the digit, or its junction with the hand. The needle is inserted, just before applying the rubber, through the skin of the lateral aspect of the dorsum of the finger, an inch from and on the distal side of the ligature. Two minims are ejected, the needle pushed on one-fourth of an inch further, two minims more forced out, and so on, until the point rests under the skin on the plantar aspect of the digit, when a large quantity is ejected. One-half the finger is thus injected, and the operation repeated on the other half. The patient feels a smarting, burning pain, the tourniquet is tightened, and absorption awaited. Massage hastens this process. In about two minutes insensibility should supervene, and the operation begun. Repeat the injection if the anesthesia be insufficient. Fifteen minims is usually found sufficient, but thirty may be given without risk. The operation finished, the constriction is slowly and cautiously removed to prevent suddenly throwing a large amount of cocaine into the circulation. It is, therefore, let out in small quantities by loosening the band *for a minute*, when the circulation becomes restored and the wound bleeds freely, thus giving escape to the cocaine solution in the arterioles. The rubber is then tightened for two or three minutes, during which time the sutures and dressing are applied. Thus by alternately loosening and tightening the tourniquet, only a small quantity of the solution is admitted toward the heart and nerve centers, which thus become gradually accustomed to its presence. The advantages of the *direct* method are: (1) The rapidity of the anesthesia (practically instantaneous); (2) the small quantity of cocaine employed; (3) the escape of a good part of the injected solution through the wound of incision. Either one or the other of these methods is so generally applicable, that almost all the surgery of the fingers and toes can be performed under cocaine anesthesia. When more than one digit is involved, the large quantity of cocaine necessary might endanger the patient, and here general narcosis is to be preferred. The author then proceeds to enumerate various operations in which cocaine can be used as the anesthetic.

Among these are operations in Dupuytren's contraction, palmar phlegmon, suturing of tendons, limited bone removal, extirpation of ganglia at wrist, and all other kinds of smaller neoplasms, and all minor operations upon the legs, thighs, forearms and arms. When more than one drachm of the solution is used, Professor Wyeth recommends injecting a limited quantity, and then operating in the line of the injection, thus giving escape to a good proportion of the solution used.

Operations on the trunk require greater care, because of the immediate absorption of the solution. The author takes the removal of a fatty tumor for an example, and advises as follows:

> In the proposed line of incision carry the needle point into the deep layers of the skin (not into the subcutaneous fat, for it is desired to reach the end organs of the sensory nerves in the papillary layer), and force out one-half to one minim, advance the needle a fourth of an inch, and repeat the injection, and so on as far as the needle will reach from the original puncture. Re-insert the needle and repeat the method just described, until the line of anesthesia is established. In this manner an incision as long as three inches can be made without pain, by the judicious distribution of from ten to fifteen minims of a four per cent. solution.

Pallor of the skin indicates anesthesia, which is almost instantaneous. Incision is made in the middle of the anesthetized line, and continued laterally until pain is experienced, which may be an inch or more on either side. The operation is proceeded with by half a minim, or more, at all sensitive points, and working directly in the line of injection. Thus may be removed fatty tumors, three or four inches in their longest measurement, cystic tumors, two inches or less in diameter, all forms of neoplasms, angiomata, moles, cicatrices, etc., not covering a larger area than four square inches. Superficial foreign bodies can be removed and abdominal wounds explored down to, but not through, the parietal peritoneum. Lesions of the scalp can also be treated. Operations upon the face and neck, where scars are objectionable, and where accurate apposition of the edges requires more time than cocaine anesthesia will insure, can often be better done under general anesthesia. However, moles, warts, naevi, scars, impacted bodies, etc., can be removed with cocaine.

In the surgery of the eye, cocaine enjoys a wide range of applicability, one or two drops in the conjunctival sac, repeated every minute for from two to five minutes, allows extraction of foreign bodies, applications to the conjunctiva and cornea, removal of pterygium, pinguecula, polypus, lithiasis, incision, scraping or cauterization of ulcers of the cornea, sections of the cornea, and sclero-cornea for cataracts, including all kindred operations. Squint and minor operations upon the lids and lachrymal apparatus can also be performed under cocaine anesthesia.

Diseases of the buccal cavity, such as tumors, one-half to one inch in diameter, cysts of the lips, chronic fissures or ulcers, adhesions, etc., may be treated under cocaine. Small epitheliomata, or suspicious ulcers, are made insensible by injecting 5-20 minims of a four per cent. solution beneath and around their base. Small ranulæ, and complete cleft of the small palate, can also be operated upon painlessly in this way.

In laryngeal, nasal and naso-pharyngeal surgery, cocaine is widely used every day.

In the surgery of the genito-urinary organs, cocaine has found a field of great usefulness. The method of use prescribed by the writer is as follows, viz.:

> After disinfection of the male urethra with boracic acid solution (gr. x.—℥i) from one to two drachms of a four per cent. cocaine solution are injected by the ordinary P syringe. The canal should be fairly distended by grasping the meatus with the thumb and finger, closing it so that the mucous membrane may be brought in contact at all points with the solution.

The excess is allowed to escape. If the membranous urethra be the seat of operation, a deep injection of twenty to thirty minims must be made. Over-distension is to be avoided, else the wound may gape and the solution be forced into the circulation. Not over a drachm should be first injected. One to two minutes secures anesthesia, examination is made, and the excess of cocaine solution washed out with a solution of boracic acid.

The author finds cocaine likewise useful in operations for circumcision in adults, hydrocele, hemorrhoids, single and superficial fistulæ, and ends by expressing his belief that "cocaine surgery has not yet received its deserved appreciation." J. P.

THE English courts have lately decided that in a case "where a wound is given, which, in the opinion of competent medical advisers, is dangerous, and the treatment which they adopt is the immediate cause of death, the party who inflicted the wound is criminally responsible." This decision was reached in a case in which it was sought to shift the responsibility from the persons who inflicted the wound, upon the doctors, who sought to save the man's life. Thus the surgeon is free from more than ordinary responsibility in treating such cases. As a result, he will be inclined to undertake operations that otherwise he would not, and so give the accused a better chance of avoiding the charge of murder.—*Maryland Medical Journal.*

